# A modified culture medium and hyphae isolation method can increase quality of the RNA extracted from mycelia of a dimorphic fungal species

**DOI:** 10.1007/s00294-021-01181-4

**Published:** 2021-04-10

**Authors:** László Attila Papp, Lajos Ács-Szabó, Szilárd Póliska, Ida Miklós

**Affiliations:** 1 Department of Genetics and Applied Microbiology, Faculty of Science and Technology, University of Debrecen, Egyetem tér 1, 4032 Debrecen, Hungary; 2 Department of Biochemistry and Molecular Biology, Faculty of General Medicine, University of Debrecen, Debrecen, Hungary

**Keywords:** Gelatin medium, RNA extraction, Hyphae, Dimorphism, Mycelial growth, *Schizosaccharomyces japonicus*

## Abstract

The capability of RNA isolation with good efficiency and high quality is essential for a downstream application such as RNA sequencing. It requires successful cell culturing and an effective RNA isolation method. Although effective methods are available, production of the homogenous mycelia and extraction of good-quality mycelial RNA from true invasive hyphae, which penetrated into the agar plates, are difficult. To overcome these problems, the aim of this study was to develop technical modifications which allow production of homogenous mycelial biomass without extra stimuli agents and improve quality of the RNA extracted from the fungal hyphae. Our alternative culture medium was suitable for production both yeast-phase cells and hyphae of the *Schizosaccharomyces japonicus* and other dimorphic species, such as the *Candida albicans, Saccharomyces cerevisiae,* and *Jaminaea angkorensis.* To improve quality of the mycelial RNA, we developed an isolation procedure of the hyphal tip, which eliminated the unnecessary vacuoles-containing parts of the hyphae. To increase RNA quantity, we used glass beads in the RNA extraction protocol to achieve stronger breaking of the mycelial walls. All these modifications can also be useful for researchers working with other dimorphic fungi and can contribute to the higher comparability of the transcriptional data coming from yeast-phase cells and hyphae or even from different species.

**Supplementary Information:**

The online version contains supplementary material available at 10.1007/s00294-021-01181-4.

## Introduction

High-quality RNA is essential for several downstream applications, including RNA sequencing, which is an attractive method for the transcriptional profiling. To achieve good-quality RNA, in general, we need the proper amount of yeast cells or hyphae and a proper RNA isolation method. Although effective methods are available, several experimental problems can occur, especially when we want to extract RNA from true invasive hyphae, which penetrated the agar. The problems can occur both in producing the homogenous mycelial biomass and in extracting RNA from the hyphae. We faced these experimental problems when we wanted to carry out a transcriptional profiling analysis to reveal the dimorphic genes of the fission yeast, *S. japonicus.*

Dimorphism is a common ability of the pathogenic fungi, which can cause serious human infections or agricultural damage (reviewed in Gauthier 2017; Brand 2012; Pukkila-Worley et al. 2009; Kuo et al. 2014; Boyd et al. 2017). This capacity enables them to switch between unicellular (yeast) and filamentous (hyphae) forms (Bastidas and Heitman 2009; reviewed in Chen et al. 2020; Kim et al. 2000; Gimeno et al. 1992; Orlowski 1991; Sipiczki 1998a, b). Furthermore, the filamentous form is often critical for their pathogenesis, as suggested by the mutants, in which failure to form mycelia is associated with an avirulent phenotype (Lo et al. 1997).

Transcriptional profile analysis is a widely used method for identifying the genes involved in the filamentous growth of dimorphic species (Nantel et al. 2002; Carlisle and Kadosh 2013; Epp et al. 2010; Pomraning et al. 2018; Harcus et al. 2004; Wu et al. 2016; Bekker et al. 2011, Gomez-Gil et al. 2019). In these cases, the transcriptomic data of wild-type yeast-phase cells are compared to those obtained from mutant yeast-phase cells or hyphae. In the case of comparison of wild-type and mutant yeast-phase cells, the same culture medium can be used, which makes their culturing simple. In contrast, when mycelial and yeast-phase RNAs are necessary for the experiment, the same culture medium is not suitable for culturing of both phases. To produce sufficient amount of homogenous mycelial biomass, mycelial growth is generally induced by stimuli factors, such as heat or Fetal Bovine Serum (FBS), while the yeast-phase cells are cultured on media prepared without stimuli agents (Wu 2016; Epp 2010; Nantel et al. 2002). Since the different composition of the media and application of stimuli factors can induce different gene sets and transcriptional pathways (reviewed in Biswas et al. 2007; Nantel et al. 2002; Harcus et al. 2004; Pataki et al. 2017), the data obtained from RNA samples extracted from yeast-phase cells and hyphae grown under different circumstances are difficult to compare. In addition, further complicate comparison of the experimental data is the fact that quality and composition of the FBS can vary from lot-to-lot and they can have different inducing capacity (own experiences, Zheng et al. 2006).

Thus, one of the aims of this study was to find a common medium, which is suitable for production both of hyphae-phase and yeast-phase cells in the *Schizosaccharomyces japonicus* without addition of stimuli agent to the medium for mycelial growth*.* Besides, since RNA isolation from the hyphae is often difficult, has low efficiency, and requires altered methods (Ma et al. 2016; Shu et al. 2014; Schumann et al. 2013), we wanted to develop an easy and cheap method, which improves quality of the RNA extracted from the true invasive hyphae.

Our results suggest that the complete medium solidified with gelatin instead of agar was suitable for production both hyphae-phase and yeast-phase cells. Furthermore, this alternative medium can be used for other dimorphic species, too, such as *Candida albicans, Saccharomyces cerevisiae,* and *Jaminaea angkorensis*. We determined the optimal gelatin concentration of the medium and also developed a hyphal-tip isolation procedure combined with a tiny modification of the RNA extraction protocol, which improved quality of the mycelial RNA.

## Materials and methods

### Strains and media

Wild-type *Schizosaccharomyces japonicus* strain obtained from the Czechoslovak Collection of Yeasts (CCY-44-5-1, ATCC10660, CBS 354, our collection number 7-1), *Candida albicans* (SC5314, ATCC MYA-2876), *Saccharomyces cerevisiae* (10–642), and *Jaminaea angkorensis* (CBS 10918, CCY 88-1-1, 11-188) (Sipiczki and Kajdacsi 2009) were used in this study.

We used yeast extract, glucose, agar (YEA) medium: 2% d-glucose (Sigma-Aldrich), 1% yeast extract (Scharlau), 2% agar powder (Sigma-Aldrich), or liquid YEL (YEA prepared without agar) to culture the hypha- and yeast-phase cells. The YEL liquid medium was prepared without and with 10% FBS supplementation (Papp et al. 2014).

Besides, a modified version of YEA (called YEG) was also used, where the agar was replaced by gelatin (VWR). This altered medium was also prepared with commercially available gelatin (Oetker). This kind of gelatin was also suitable for our purposes; however, strength of the media varied from batch to batch.

### Determination of the optimal gelatin concentration and melting time of the gelatin cubes

To find the optimal gelatin concentration, we prepared YEG media with different amounts of gelatin (4–15%) and set them to different pH values by ccHCl (pH 6.8–7.0), because these factors can influence strength of the medium. Time necessary for the solidification of the media was determined by a lab timer, while hardness of the media was tested by streaking the cells onto the surface of the YEG plates.

Time necessary for melting of the small gelatin cubes at 37 °C, which were cut out from 10 and 15% gelatin media, was determined by a lab timer.

### RNA extraction

We used the hot phenol RNA extraction method with slight modifications (Lyne et al. 2003). Only hyphae tips were used for RNA isolation instead of whole hyphae. The hyphae tips were washed with DPEC water before RNA extraction and glass beads were added to them (200–300 µm Sigma) (100–150 µl, cc. 1/3 volume of the hyphae tips) to increase breaking of the hyphae and the yield of RNA. Later, we followed the protocol with one exception. We skipped the chloroform:isoamyl alcohol step, and immediately after the 1 h incubation, we precipitated the RNA. 100 µl DEPC water was added to the samples and they were stored at – 70 °C.

### RNA quality check

Quality of the total RNA samples was checked on Agilent BioAnalyzer using Eukaryotic Total RNA Nano Kit, according to manufacturer’s protocol. In the gel electrophoresis, 5–5 µl RNA samples were run on 1% agarose gel, in 1 × TBE buffer.

### Morphology of the cells and colonies

Cell morphology was investigated under an Olympus BX40 microscope, while the mycelia were studied under a Carl Zeiss Jena Stereomicroscope.

### Statistical analyses

Normal distributions of the data were tested using the Shapiro–Wilk test. Since our datasets proved to be normally distributed, two-sample *t* test was used for statistical evaluation. *P* values were considered significant below the alpha level 0.05. Statistical analyses were performed in PAST v.3.20 software (Hammer et al. 2001).

## Results

### The gelatin containing medium enabled production of hyphae and cell division of the yeast-phase cells

**Fig. 1:**
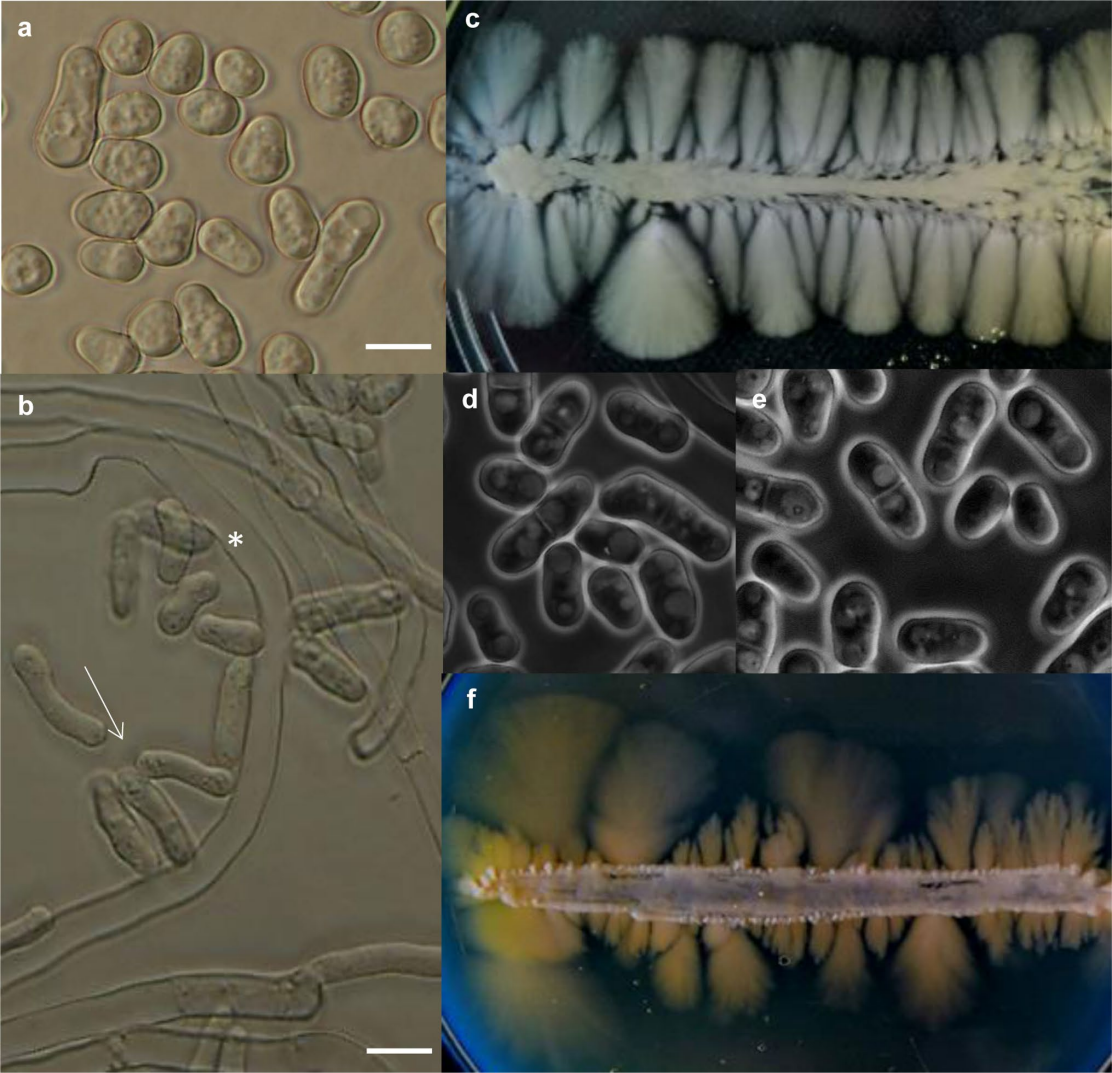
Hyphal growth and cell morphology on different media. *S. japonicus* could not produce mycelia in liquid YEL medium (**a**) (37 °C, overnight). The hyphae production required at least 10% FBS supplementation (**b**) (37 °C, overnight). The FBS containing YEL medium always contained mixture of long hyphae (indicated with asterisk) and yeast-phase cells (indicated with white arrow). The standard agar plates (YEA) (**c**) and the medium solidified with gelatin (**f**) allowed production of hyphae and yeast-phase cells (**d**, **e**). The Perti dishes were incubated at 30 °C. The hyphae were photographed after 10 days, while the yeast–phase cells after 2 days. **a**, **b**: Nomarski microscopy, **d**, **e**: phase–contrast microscopy. Bar: 10 μm

To find a common medium suitable for producing both the yeast-phase and hyphae-phase cells, we tested different media. The liquid medium (YEL), from which mycelia could easily be removed, was not suitable, because hyphae production could be achieved only in the presence of Fetal Bovine Serum (FBS) (Fig. 1a, b) and even this stimulus-containing medium resulted in a mixture of hyphae and yeast cells and not a homogenous mycelial culture (Fig. 1b). Thus, later the solid media were tested. The 2% agar containing medium (YEA), which was used as a standard medium, enabled growth of the mycelial-phase and yeast-phase (Fig. 1c, d), but it did not favour isolation of hyphae, which penetrated into the agar, and extraction of the high-quality RNA (see it later). That is why we wanted to prepare another solid medium, which is softer than the agar plates. Since earlier, hyphae embedded in gelatin have successfully been used for photography (Sipiczki et al. 1998a, b), we prepared culture media solidified with gelatin.

To find the optimal gelatin concentration, media with different amounts of gelatin (4–15%) were prepared. Our results showed that all media became solid at room temperature (media containing 4–5% gelatin solidified after 20–25 min, while the 10–15% gelatin media solidified after 10–12 min), but, as we expected, their hardness was different. Our tests revealed that the 4–5% gelatin media were too soft at 30 °C (it was used as an incubation temperature), while the 10 and 15% gelatin media (pH 6.8) had proper strength. They were suitable for streaking or spreading of the cells on their surfaces. The later media enabled production of both the yeast-phase cells (Fig. 1e) and hyphae (Fig. 1f).

### An alternative hyphae isolation procedure from the gelatin medium for RNA extraction

**Fig. 2:**
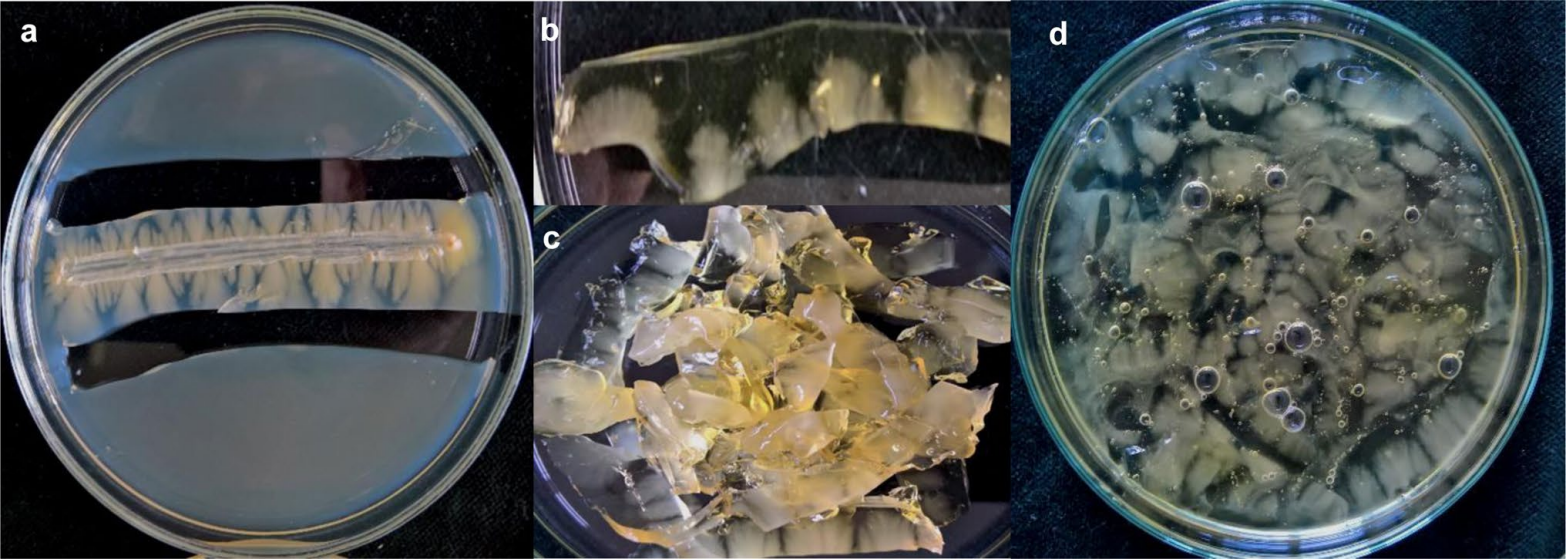
Steps of isolation of the hyphal tips for RNA extraction. The hyphal tips were cut out from the agar and gelatin plates (**a**). The agar and gelatin cubes were collected into sterile petri dishes (**b**). The cubes were chopped into smaller pieces (**c**). The agar–hyphae cubes were immediately submitted to RNA extraction, while the gelatin–hyphae cubes were incubated for 5 min at 37 °C to melt the gelatin (**d**). The molten gelatin–hyphae mixture was centrifuged and the gelatin (the upper phase) was removed. The hyphal tips (pellet) were used for RNA isolation

Since the solid media YEA (solidified with agar) and YEG (solidified with gelatin) enabled production of the hyphae-phase and yeast-phase cells (Fig. 1), we streaked the *S. japonicus* cells onto the surface of these media, and after 10 days, we isolated the hyphae. Since the *S. japonicus* mycelia were mainly located under the surface of the medium, we tried to “dig them out” from the YEA and YEG media, but this procedure resulted in broken hyphae and low-quality RNA. Furthermore, the long hyphae of *S. japonicus* always contained a large number of vacuoles (Fig. S1), whose content could also contribute to RNA degradation. Therefore, we developed a hyphal-tip isolation procedure, in which only the tips of hyphae were cut out from the agar and gelatin plates (Fig. 2a). The small agar and gelatin cubes were collected (Fig. 2b) and chopped into smaller pieces (Fig. 2c). To separate the hyphae from the agar and gelatin, we tried to melt these agar- and gelatin cubes. Melting of the agar cubes required a high temperature (above 65 °C) and longer incubation time, which is why we later used the agar–hyphae cubes without melting for the RNA extraction. At the same time, the gelatin cubes could be melted at 37 °C (Fig. 2d) (since melting time of the 10% gelatin containing cubes was shorter (5 min) than the time necessary for melting of the 15% gelatin cubes, we used the 10% gelatin plates for the production of mycelia). The hyphal tips were separated from the melted gelatin with centrifugation and were immediately used for RNA isolation (for details, see the supplementary material: step-by-step protocol).

### RNA extraction from the hyphal tips

**Fig. 3:**
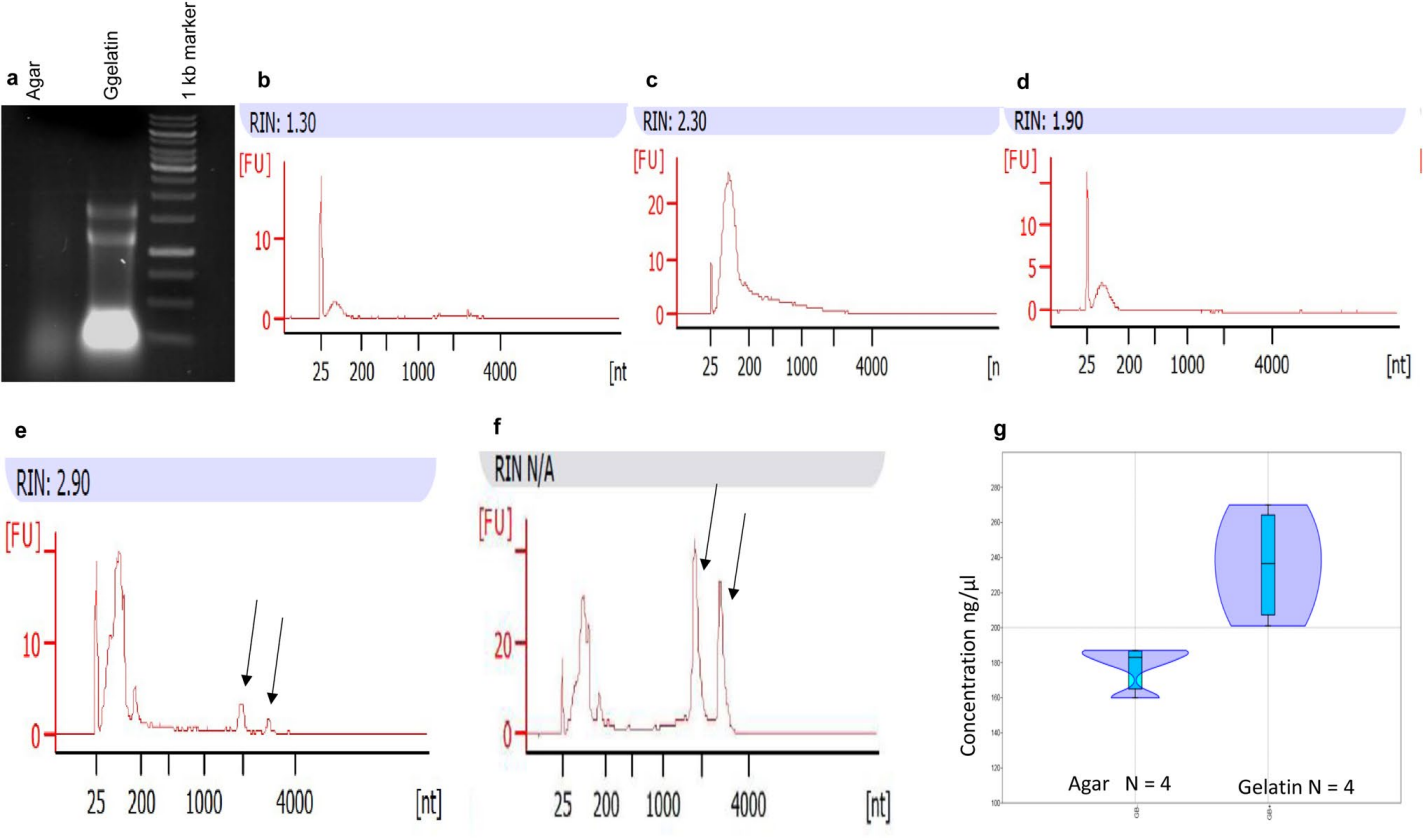
Quality check of the RNA isolated from hyphae. Agarose gel electrophoresis of the RNA isolated from agar and gelatin plates (**a**) Electropherograms of RNA extracted from sheared agar–hyphae pulp (**b**), from intact agar slices without (**c**) or with glass beads (**d**). 18 and 28S RNA peaks are missing. Electropherograms of the RNA samples isolated from gelatin medium without (**e**) and with glass beads (**f**). 18 and 28S RNA peaks appeared (indicated by black arrows). RIN: RNA integrity number. Distributions of the different RNA concentrations isolated from the hyphal tips separated from agar and gelatin (**g**). Violin plots show the kernel density for each samples. Box plots in the violin plots indicate the 25–75% quartiles. Horizontal lines within the boxes show the medians of the samples. Minimal and maximal values are depicted by the whiskers. Concentrations shown in the *Y*-axis are tenfold dilutions of the original samples. *N*: sample size. The values of the samples were significantly different (two-sample *t* test, *P* = 0.011261). The data indicated that the quantity of the RNA isolated from the gelatin plates were higher than the quantity of RNA isolated from the agar plates

To isolate RNA samples necessary for RNA sequencing, we followed the hot phenol RNA extraction protocol (Lyne et al. 2003), with some modifications (see in the methods and in the supplementary material: step-by-step protocol). Quality of the RNA samples was investigated both by Agilent BioAnalyzer and gel electrophoresis method. The quality check showed that the RNA isolated from the agar–hyphae cubes was degraded (Fig. 3a) and the 18 and 28S RNA peaks were missing, independent of the alterations of the RNA extraction method [the initial sample was shared agar–hyphae pulp (Fig. 3b), intact agar–hyphae cubes without glass beads (Fig. 3c) or with glass beads (Fig. 3d)]. The quality assay showed that RNA samples extracted from the agar cubes were simply not suitable for RNA sequencing. In contrast, yield and quality of the RNA isolated from those hyphal tips which were separated from the melted gelatin–hyphae cubes were higher (Fig. 3a) and the 18 and 28S peaks, which indicate quality of the RNA, could also be found in it (Fig. 3e, f). The addition of the glass beads to the hyphal tips clearly improved both quality (Fig. 3f) and quantity of the RNA (Fig. 3g). This RNA sample was successfully used for RNA sequencing, as proved by the heat map, which showed a selected gene set of the transcriptional profiling analysis (Fig. S2). Further data of the transcriptional profiling analysis can be found in a separate article.

### The gelatin medium enabled the mycelial growth of other dimorphic species

**Fig. 4:**
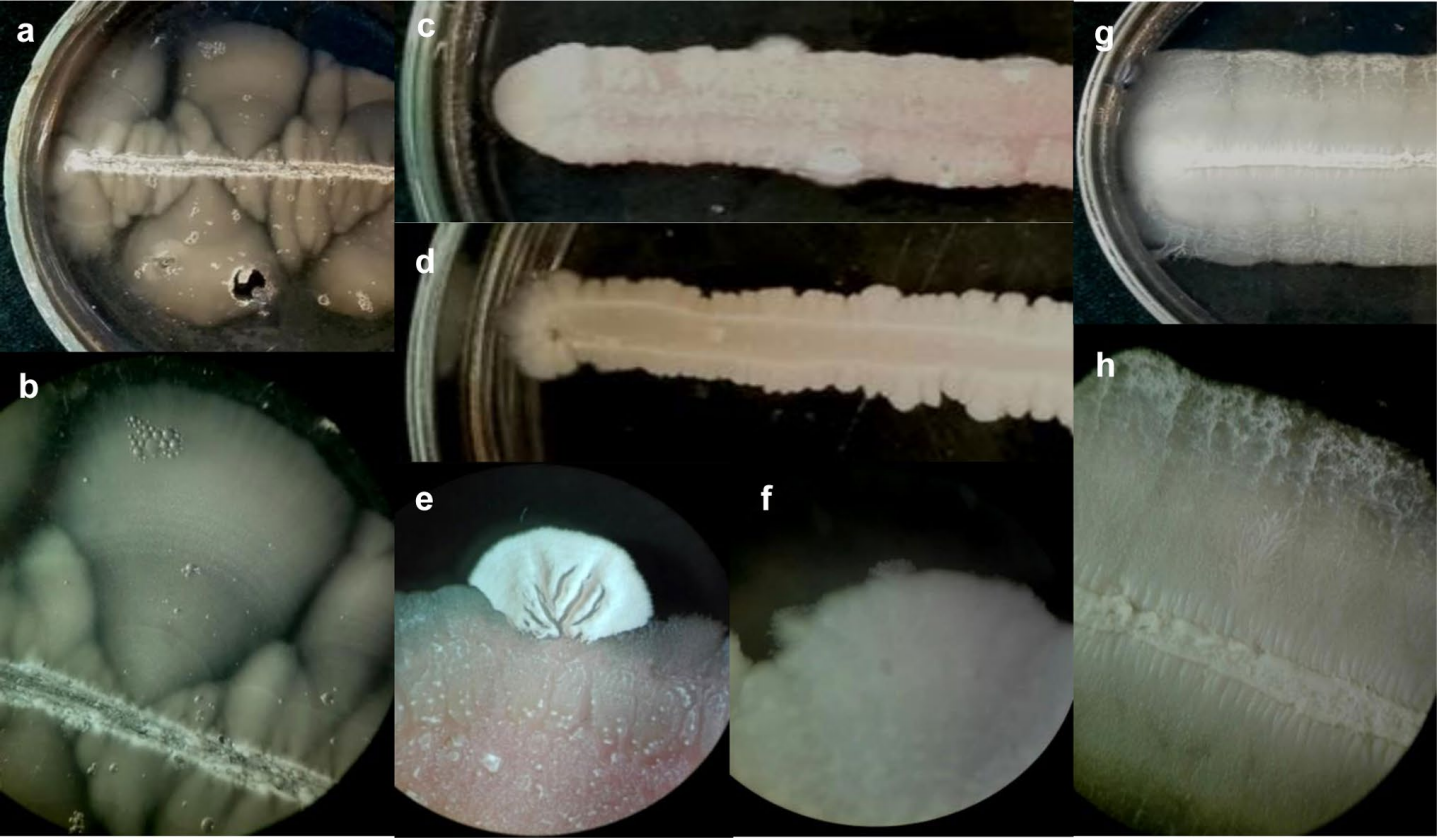
Mycelial growth on 10% gelatin containing medium (YEG*). S. japonicus* (**a**, **b**) *J. anghorensis* (**c**, **e**) *S. cerevisiae* (**d**, **f**), (*C. albicans* (**g**, **h**) incubated at 30 °C for 7–10 days). **b**, **e**, **f**, **h** are stereo microscopic photos

We wanted to know whether or not the gelatin medium can be used in case of other dimorphic yeast species. To this end, the dimorphic *Saccharomyces cerevisiae*, *Candida albicans,* and *Jaminaea angkorensis* were streaked out onto the surface of 10% gelatin media (YEG) and incubated at 30 °C for 10 days. All the three species could form hyphae on this medium (Fig. 4).

## Discussion

Transcriptional profiling is a useful method to reveal the molecular background of mycelial growth of fungi (Nantel et al. 2002; Harcus et al. 2004; Wu et al. 2016). This method requires proper amounts of hyphae and yeast-phase cells (which are used as control) and high-quality RNA extracted from yeast cells and hyphae. Despite the availability of culturing and RNA extraction methods, we faced several experimental problems in our efforts to prepare RNA samples from the *S. japonicus* invasive hyphae for RNA sequencing. The first difficulty was production of homogenous mycelial biomass, because, like other dimorphic species, the *S. japonicus* did not form hyphae in liquid medium without application of stimuli agents (Sipiczki et al. 1998b; Papp et al. 2014; Nantel et al. 2002; Harcus et al. 2004).

Composition of the medium can seriously influence which genes function, at least this is what the transcriptional data suggest which come from the yeast-phase cells or hyphae which grown under different circumstances (reviewed in Biswas et al. 2007; Nantel et al. 2002; Harcus et al. 2004; Pataki et al. 2017). Consequently, we wanted to avoid addition of external stimuli agents to the medium for hyphae. Thus, the liquid medium could not be used for production of hyphae. Similarly, we could not use the standard agar plate either, because although it was suitable for production of the mycelial biomass, it was too hard to isolate back the true invaded hyphae from it. To overcome these problems, we prepared a modified solid medium (YEG, solidified with 10% gelatin), which allowed production the mycelial biomass without addition of inducing agents, and separation of the hyphae from the medium. Furthermore, this medium also allowed mycelial growth of the distantly related dimorphic species, such as *S. cerevisiae*, *C. albicans* and *J. angkorensis*. These species did not hydrolyze gelatin, unlike *Paracoccidioides* (Bedoya-Escobar et al. 1993), their yeast-phase cells divided well and they also produced hyphae, suggesting that this altered medium could be applied in other dimorphic species, too. However, the longer incubation time of hyphae could lead to depletion of some components of the culture medium and to activation of quorum sensing mechanisms (Sipiczki et al. 1998b; Gomez-Gil et al. 2019). In spite of this fact, we believe that application of the same initial medium for the yeast-phase cells and mycelial phase, is better than application of two different media or different culture conditions.

Our further technical modifications concerned isolation of the hyphae from the solid medium and the RNA extraction. These alterations became necessary, because quality of the RNA isolated from the whole hyphae using the traditional method was low and unsuitable for RNA sequencing (Lyne et al. 2003).

We assumed that quality problems of the mycelial RNA could be related to the fact that *S. japonicus* hyphae had invaded the medium and contained a large number of vacuoles (Sipiczki et al. 1998a,b; Papp et al. 2014; Kinnaer et al. 2019). Consequently, when we isolated mycelia from the medium, they necessarily were broken and content of their vacuoles might have contributed to degradation of the RNA extracted. This assumption requires further study, but it can be in good agreement with the findings that degradation of the nuclear components may be associated with macroauthophagy (Shoji et al. 2010). To overcome these problems, we developed a protocol which enabled us to isolate the cytoplasm-filled hyphal tips, but the vacuoles were eliminated. This protocol, combined with some modifications of the hot phenol extraction method (Lyne et al. 2003) (application of glass beads to break the hyphal wall) contributed to the higher yield and better quality of the RNA obtained from hyphae.

In summary, we believe that our altered culture medium, the hyphal-tip isolation procedure and modification of the RNA extraction method can also be useful for researchers, who are working with other dimorphic fungi and want to extract RNA from true invaded mycelia. All these modifications can also contribute to the better comparability of the transcriptional data which come from yeast-phase cells and hyphae or possibly different species.

## Supplementary Information

Below is the link to the electronic supplementary material.

Supplementary file1 (DOCX 17 KB)

Supplementary file 2 Fig. S1*S. japonicus* mycelia contained a large number of vacuoles (YEL+10% FBS, at 37 °C, overnight.) Hyphal-tip (a) (indicated with white star), vacuoles in the hyphae (b) (vacuoles are indicated with white arrows). (PPTX 545 KB)

Supplementary file 3 Hierarchical clustering analysis of the selected genes that are differentially expressed in thehyphae compared to the yeast cells. The RNA samples were isolated from the *S. japonicus*yeast cells and hyphae grown on YEG medium at 30°C, for 1 day (yeast cells) and for 10 days(hyphae). Value1: average log2 fold-change values of yeast cells, Value2: average log2 foldchangevalues of hyphae. The data came from three separate experiments. (PPTX 122 KB)

## Declarations

### Availability of data and materials

All data generated or analysed during this study are included in this published article and its supplementary information files.
