# Supplementary material for: A modified culture medium and hyphae isolation method can increase quality of the RNA extracted from mycelia of a dimorphic fungal species
Source: Curr Genet. 2021 Apr 10;67(5):823–30. doi: 10.1007/s00294-021-01181-4 (PMC8405466; doi:10.1007/s00294-021-01181-4)
Supplement: Supplementary file 1 — Supplementary file1 (DOCX 17 KB) [file 294_2021_1181_MOESM1_ESM.docx]

Hyphae tip isolation procedure and RNA extraction:

1. streak the yeast cells in the middle of the YEG (2% D-glucose, 1% yeast extract (Scharlau), 10% gelatin) plates with sterile inoculation loop
2. incubate the plates at 30°C for 10 days *
3. excise the tips of the growing hyphae with sterile scalpel
4. transfer the slices to a new, sterile petri dish **
5. incubate the slices at 37°C for 5 min ***
6. carefully transfer the melted gelatin-hyphae suspension to empty 2ml Eppendorf tube(s) up to 500 µl
7. add immediately room temperature DEPC water up to 2 ml and suspend it. This step will dilute the gelatin and prevent to re-solidify
8. centrifuge the tubes for 1 min at 2000 rpm, then discard the supernatant
9. wash the hyphae with 1 ml ice-cold DEPC water
10. centrifuge the tubes for 1 min at 2000 rpm
11. repeat step 8) and 9) for two times
12. prepare 2 ml Eppendorf tubes with 100-150 µl glass-beads
13. add 750 µl TES solution to the pelleted hyphae then transfer the suspension to the Eppendorf tube with glass-beads
14. add immediately 750 µl acidic phenol to the suspension and vortex the tubes briefly
15. incubate the tubes at 65°C in dry/water block for 1 hour
16. vortex the tubes for 10-15 seconds in every 10 minutes
17. chill the tubes on ice then vortex for 15-20 seconds
18. centrifuge the samples at 10000 rpm for 10 minutes at 4°C
19. transfer the water phase to a new 2 ml Eppendorf tube then add equal amount of acidic phenol then vortex the tubes for 15-20 seconds

Optional: use phase-lock tubes (MaXtract High Density, Cat. No:129056) to efficiently eliminate the phenol (DO NOT VORTEX THE TUBES)

1. centrifuge the samples at 10000 rpm for 10 minutes at 4°C
2. transfer the water phase to a new 2 ml Eppendorf tube then add 0.5 volume Na-acetate (3M) and 2.5 volume absolute ethanol
3. mix the tubes by inverting then incubate the samples at -20°C for 30-40 min

Pause point: incubation can be extended to overnight

1. centrifuge the tubes at 10000 rpm for 15 min at 4°C then discard the supernatant
2. add 100 µl 70% ethanol (made with DEPC water, DO NOT VORTEX)
3. quick spin the tubes (few seconds) and discard the 70% ethanol (be careful to not touch the pellet with the pipette tip)
4. dry the pellet in sterile box
5. add 100 µl of DEPC water to the pellet and incubate the tubes at 65°C for 1 min

Store RNA samples at -70°C

TES solution: (Lyne et al. 2003).

- 10 mM TRIS-HCl pH:7,5
- 10 mM EDTA pH:8,0
- 0,5% SDS

Use DEPC treated water to make all your solutions.

* the incubation time depends on the strain/species

** in case of low amount of slices the Eppendorf tubes is better than petri dishes

*** gelatin slices will quickly melt at 37°C, but the time depends on the amount of slices and the percentage of gelatin in the media

Materials and equipment:

- Eppendorf tubes (2ml)
- Scalpel (or any sterile knife)
- 37°C incubator
- Dry or water block with 65°C temperature
- Vortex
- Ice
- Glass-beads (200-300 µm)
- DEPC water
- Phenol/Chloroform/Isoamyl-alcohol (25:24:1 mixture, pH:5,2)
- Ethanol (absolute)
- Sodium-acetate (3M)
