# Supplementary figures and images for: A modified culture medium and hyphae isolation method can increase quality of the RNA extracted from mycelia of a dimorphic fungal species
Source: Curr Genet. 2021 Apr 10;67(5):823–30. doi: 10.1007/s00294-021-01181-4 (PMC8405466; doi:10.1007/s00294-021-01181-4)

## Slide 1
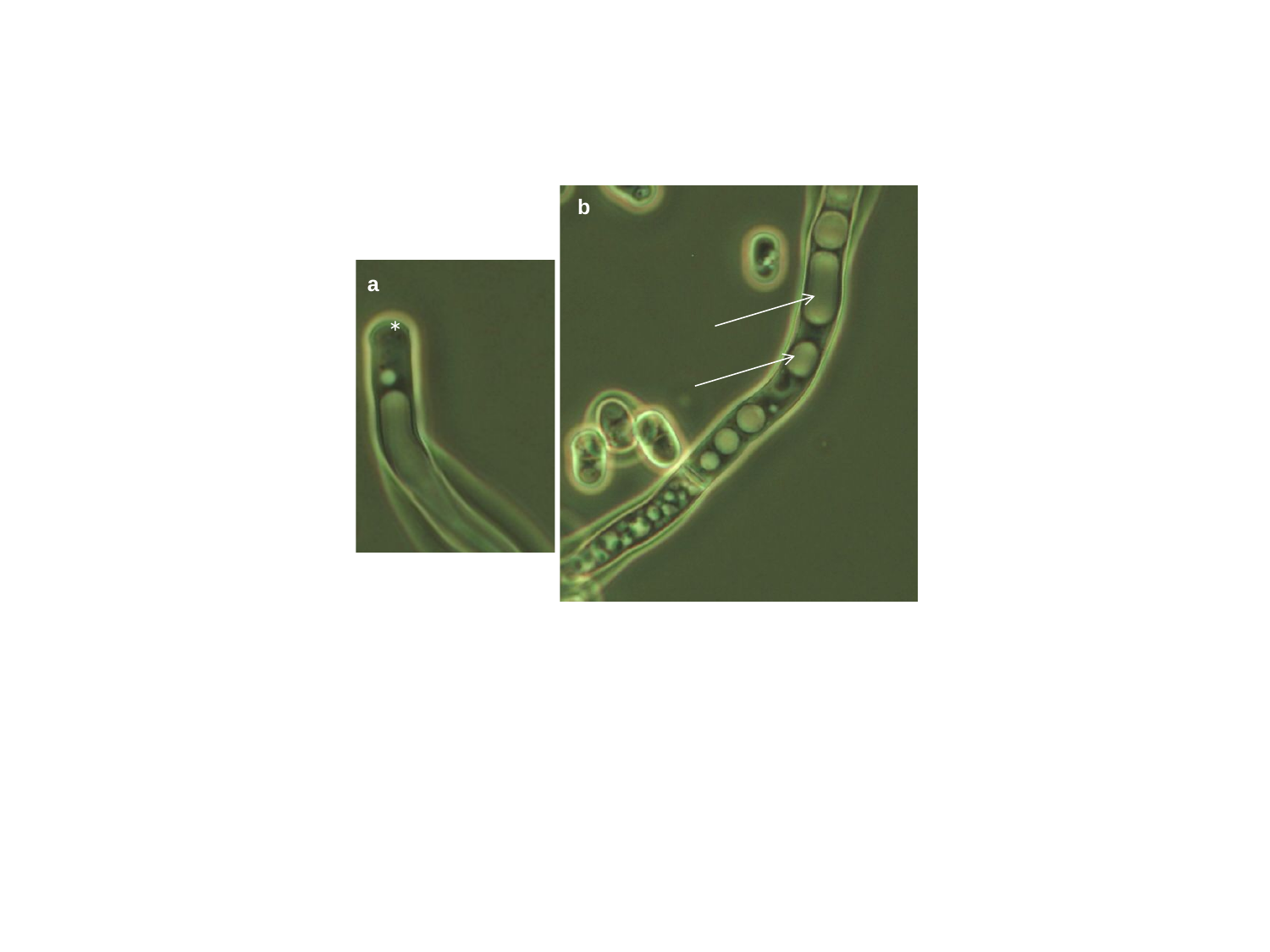

b
a
*

Supplement: Supplementary file 2 — Fig. S1S. japonicus mycelia contained a large number of vacuoles (YEL+10% FBS, at 37 °C, overnight.) Hyphal-tip (a) (indicated with white star), vacuoles in the hyphae (b) (vacuoles are indicated with white arrows). (PPTX 545 KB) [file 294_2021_1181_MOESM2_ESM.pptx]

## Slide 1
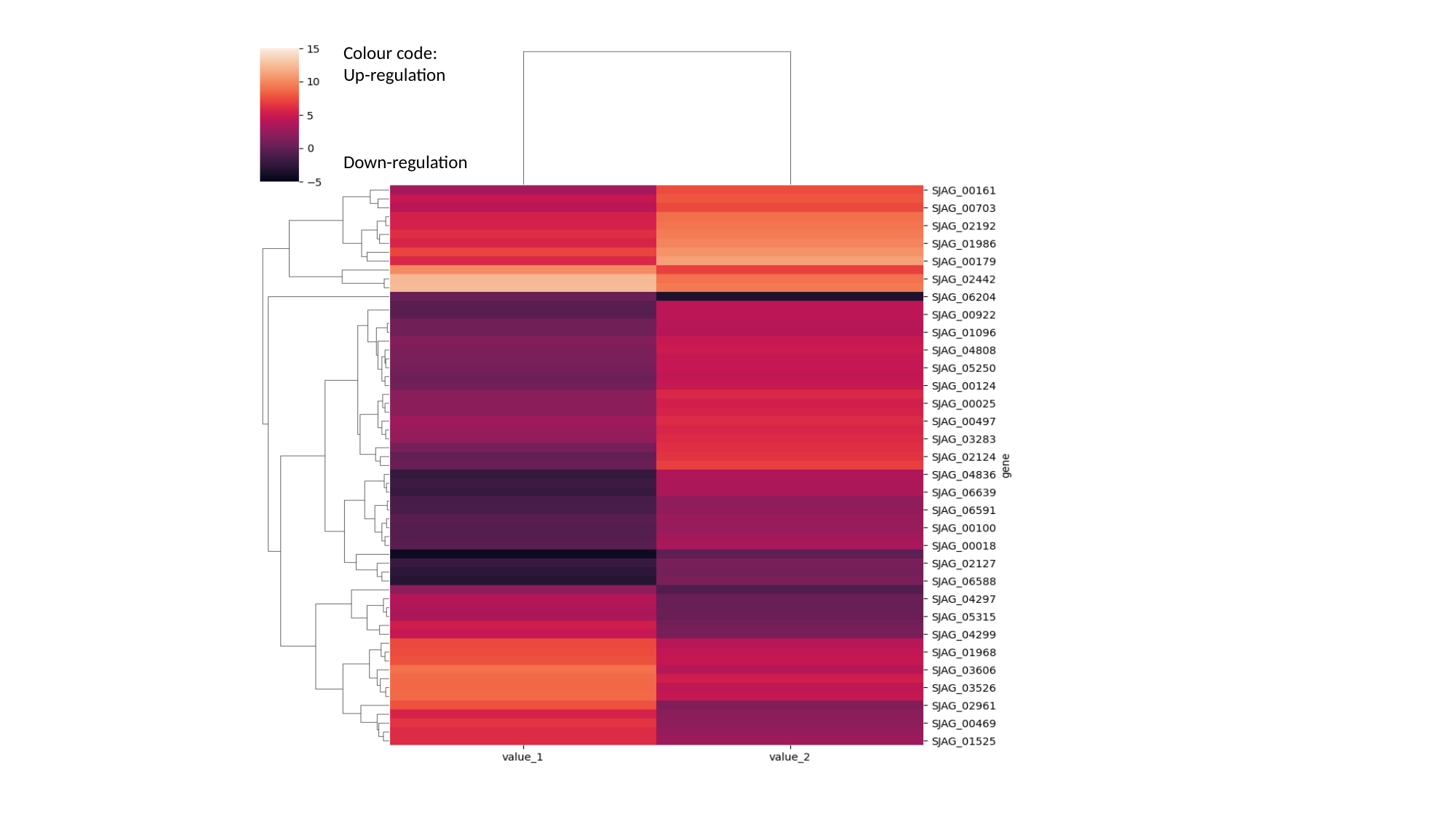

Colour code:
Up-regulation
Down-regulation

Supplement: Supplementary file 3 — Hierarchical clustering analysis of the selected genes that are differentially expressed in thehyphae compared to the yeast cells. The RNA samples were isolated from the S. japonicusyeast cells and hyphae grown on YEG medium at 30°C, for 1 day (yeast cells) and for 10 days(hyphae). Value1: average log2 fold-change values of yeast cells, Value2: average log2 foldchangevalues of hyphae. The data came from three separate experiments. (PPTX 122 KB) [file 294_2021_1181_MOESM3_ESM.pptx]
